# Ephrin-A5 Suppresses Neurotrophin Evoked Neuronal Motility, ERK Activation and Gene Expression

**DOI:** 10.1371/journal.pone.0026089

**Published:** 2011-10-11

**Authors:** Christin Meier, Sofia Anastasiadou, Bernd Knöll

**Affiliations:** Neuronal Gene Expression Laboratory, Department of Molecular Biology, Interfaculty Institute for Cell Biology, Eberhard-Karls-University Tübingen, Tübingen, Germany; University of South Florida, United States of America

## Abstract

During brain development, growth cones respond to attractive and repulsive axon guidance cues. How growth cones integrate guidance instructions is poorly understood. Here, we demonstrate a link between BDNF (brain derived neurotrophic factor), promoting axonal branching and ephrin-A5, mediating axonal repulsion via Eph receptor tyrosine kinase activation. BDNF enhanced growth cone filopodial dynamics and neurite branching of primary neurons. We show that ephrin-A5 antagonized this BDNF-evoked neuronal motility. BDNF increased ERK phosphorylation (P-ERK) and nuclear ERK entry. Ephrin-A5 suppressed BDNF-induced ERK activity and might sequester P-ERK in the cytoplasm. Neurotrophins are well established stimulators of a neuronal immediate early gene (IEG) response. This is confirmed in this study by e.g. *c-fos*, *Egr1* and *Arc* upregulation upon BDNF application. This BDNF-evoked IEG response required the transcription factor SRF (serum response factor). Notably, ephrin-A5 suppressed a BDNF-evoked neuronal IEG response, suggesting a role of Eph receptors in modulating gene expression. In opposite to IEGs, long-term ephrin-A5 application induced cytoskeletal gene expression of tropomyosin and actinin. To uncover specific Eph receptors mediating ephrin-As impact on neurotrophin signaling, EphA7 deficient mice were analyzed. In EphA7 deficient neurons alterations in growth cone morphology were observed. However, ephrin-A5 still counteracted neurotrophin signaling suggesting that EphA7 is not required for ephrin and BDNF crosstalk. In sum, our data suggest an interaction of ephrin-As and neurotrophin signaling pathways converging at ERK signaling and nuclear gene activity. As ephrins are involved in development and function of many organs, such modulation of receptor tyrosine kinase signaling and gene expression by Ephs might not be limited to the nervous system.

## Introduction

During brain development, axons encounter attractive and repulsive guidance cues, whose interplay instructs growth cones with directional information, thereby ensuring target recognition. For instance, axons initially overshoot their final termination zone until, later on, such ectopic arborizations are eliminated and only branches in the prospective termination zone are stabilized, a process termed axon pruning [1], [2]. In the hippocampus, mossy fibers are subject to axon pruning [3]. The overshooting requires growth-promoting/attractive molecules such as neurotrophins [4], whereas axon retraction involves growth-inhibiting/repulsive cues such as ephrins [5], [6], [7], [8].

Here, we studied axon guidance responses elicited by ephrin-A and BDNF co-stimulation of mouse primary neurons. Eph family members signal bi-directionally. In EphA forward signaling, ephrin-A ligands can activate multiple Eph receptor tyrosine kinase receptors (EphA1-EphA8 and also e.g. EphB2; see below) in a highly promiscuous manner. This usually results in contact-mediated repulsion, e.g. growth cone collapse [9], [10], [11]. Intracellular signal propagation via EphA receptors involves e.g. Rho-GTPases, Src and MAP kinases [9], [11], [12], [13]. In Eph reverse signaling, membrane-bound ephrin-As are “receptors” activated by EphA “ligands”. This results in attractive [14] and repulsive [15], [16], [17] axon guidance responses, depending on e.g. axonal subtype investigated. Ephrin-As such as ephrin-A5 used in this study might activate the EphB2 in addition to multiple well-established EphA receptors [18]. Therefore, ephrin-A5 activates EphA and potentially also EphB2 forward signaling (in this study summarized as Eph forward signaling).

BDNF is considered an attractive axon guidance cue, e.g. promoting retinal axon branching [16], [19], [20] and neurite outgrowth [4], [21], [22], [23]. Signaling of BDNF via the TrkB receptor results in e.g. PI3 kinase and MAP kinase activation [24]. In fact, BDNF requires MAP kinase activity to convey its impact on processes of neuronal motility as demonstrated by pharmacological inhibition of MAP kinase signaling [25], [26], [27]. BDNF modulates gene expression [24], [28], [29], which has not been reported in detail for Eph family members so far. Recently, SRF (serum response factor) emerged as transcription factor targeted by neurotrophins [30], [31], [32], [33]. SRF regulates neuronal activity-induced immediate early gene (IEG; e.g. *Egr1, c-fos, Arc*) and actin cytoskeletal gene responses [31], [34]. In *Srf* mutants, cell migration [35], neurite outgrowth, axon guidance, growth cone motility [30], [36], [37], synapse function [38], [39] and myelination [40] is impaired.

So far, an interaction of EphA forward signaling and neurotrophins has not been analyzed in detail. In contrast, a crosstalk between EphA reverse and neurotrophin signaling is well documented [16], [17], [41]. Besides neurotrophins, EphAs communicate with GDNF/Ret signaling to guide motor axons [42], [43].

Here, we demonstrate an interaction of ephrin-A and neurotrophin signaling in primary hippocampal and cortical neurons. Activation of Eph forward signaling by ephrin-A5 antagonized BDNF-enhanced neuronal motility *in vitro*. Ephrin-A5 application attenuated BDNF-stimulated ERK activity by suppression of ERK phosphorylation. Ephrin-A5 incubation resulted in co-localization of phosphorylated ERK and Eph receptors in the neurites. Eph forward signaling suppressed a BDNF-evoked IEG response conveyed by SRF. As demonstrated by EphA7 deficient neurons, EphA7 is dispensable for ephrin-A's influence on neurotrophin signaling.

Taken together, our data suggest a counteraction of neurotrophin signaling by ephrin-As. This finding might also be applicable to signaling processes regulated by Eph family members outside of the nervous system, e.g. in organogenesis and tumorigenesis.

## Materials and Methods

### Animals

*Srf (flex1neo/flex1neo)* and *Camk2α-iCre* mice were bred to obtain *Srf* mutants (*Srf^–/–^; Camk2a-iCre*) or control littermates (*Srf* ^–/–^, *Srf* ^+/–^ or *Srf* ^+/–^; *Camk2α-*iCre [35], [36]). Recombination induced by Cre recombinase expression via the C*amk2α* promoter starts just before birth and results in strong SRF down-regulation at time-points used to culture primary neurons [35], [36]. EphA7 mice were kindly provided by U. Drescher (King's College, London, UK; see [15]). Animal experiments and housing were approved by the local ethics committee (Einrichtung für Tierschutz, Tierärztlichen Dienst und Labortierkunde, Calwer Straße 7/4, 72076 Tübingen, Tübingen University; permit number: §4 Anzeige 15/10/2009).

### Neuronal cell culture

**Figure 1:**
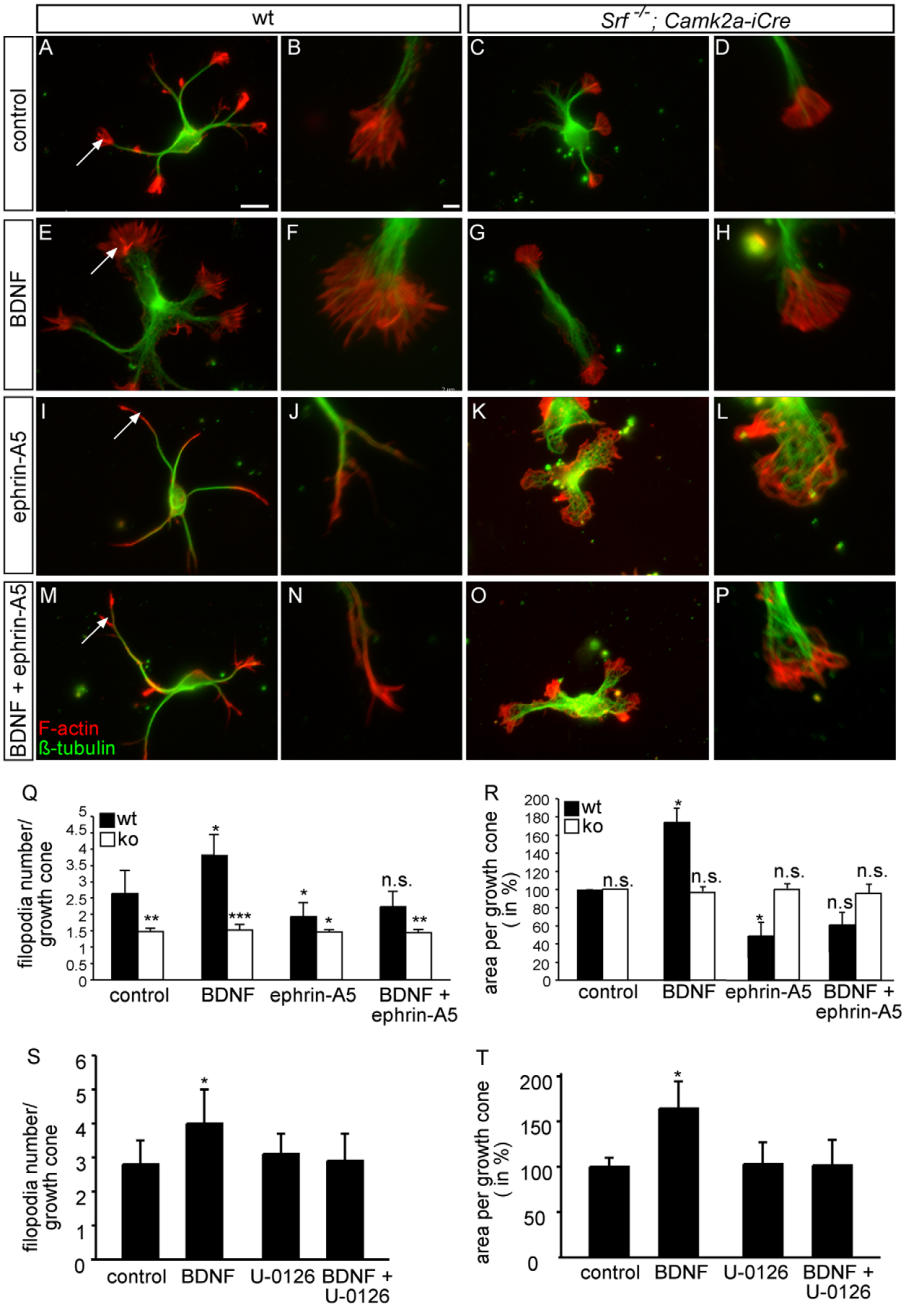
Ephrin-A5 treatment blocks BDNF-induced growth cone motility in an SRF-dependent manner. Wild-type and SRF-deficient hippocampal neurons were stimulated with ephrin-A5 and/or BDNF as indicated for 45 min, followed by F-actin (red) and microtubule (green) staining. Individual growth cones are highlighted with arrows. (A-D) Wild-type neurons protruded multiple growth cones elaborating finger-like filopodia (A, higher magnification B). In *Srf* mutants (C, D), growth cones were rounded without any obvious filopodia. (E-H) BDNF enhanced growth cone area and filopodia number in wild-type neurons (E, F). BDNF required SRF gene activity to modulate filopodia number, as revealed by *Srf* mutant neurons remaining unaltered by BDNF treatment (G, H). (I–L) Ephrin-A5 induced growth cone collapse decreasing filopodia number and growth cone area (I, J). In SRF-deficient growth cones, ephrin-A5 application resulted in ring-like structures consisting of F-actin and microtubules (K, L). (M-P) Co-application of ephrin-A5 with BDNF in wild-type neurons counteracted BDNF-stimulated growth cone motility (M, N). Now, growth cones were collapsed (M, N) rather than increased in area as induced by BDNF alone (see E, F). In SRF-deficient neurons (O, P), growth cones protruded ring-like structures as seen with ephrin-A5 alone (K, L). (Q) Quantification of filopodia number/growth cone for the various treatments and genotypes. Statistical significance was calculated relative to wild-type/control condition. (R) The average growth cone area (wild-type and control condition set to 100%) was quantified. (S, T) Upon inhibition of MEK by U-0126, BDNF fails to increase filopodia number (S) and growth cone area (T). Scale-bar (A, C, E, G, I, K, M, O)  =  10 µm; (B, D, F, H, J, L, N, P)  =  2 µm.

**Figure 2:**
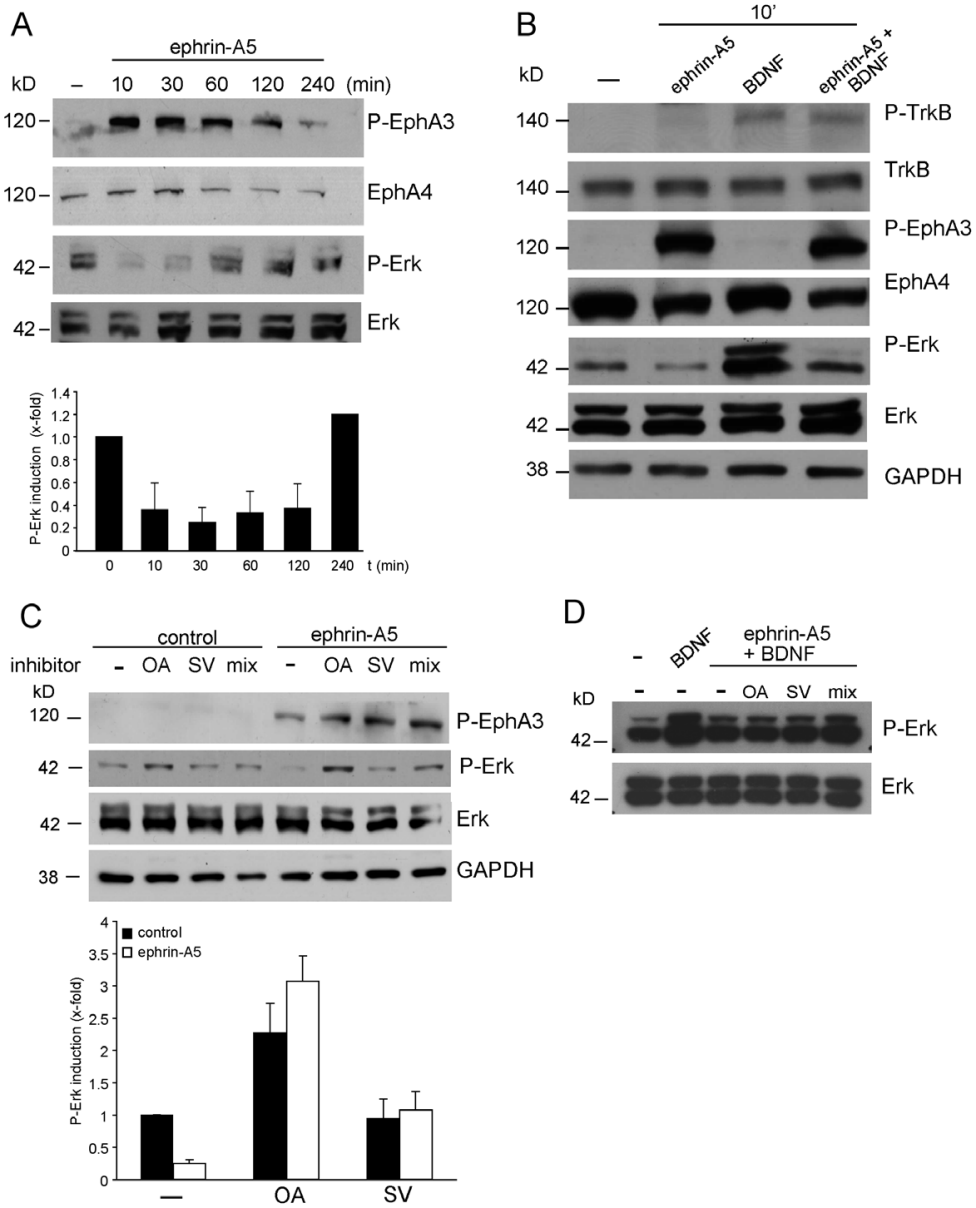
Ephrin-A5 suppresses ERK activation and inhibits BDNF-evoked ERK phosphorylation. (A) Cortical neurons were stimulated with ephrin-A5 for time-points indicated. EphA receptor activation (assessed by P-EphA3) suppressed ERK phosphorylation. At later time points (120–240 mins), EphA receptors were less active and ERK phosphorylation returned to control levels (“–”, untreated). Quantification of results depicts P-ERK levels relative to control treatment (set to1). (B) BDNF alone elevated P-ERK levels, whereas ephrin-A5 alone inhibited ERK phosphorylation. Ephrin-A5 inhibited BDNF-induced elevation of P-ERK. Neither EphA nor TrkB receptor activation was influenced by co-application of both substances. (C) Eph forward signaling requires phosphatases to suppress ERK phosphorylation. Neurons were pre-treated with phosphatase inhibitors (OA, ocadaic acid; SV, sodium vanadate; mix, commercially available phosphatase inhibitor mix). In the presence of either phosphatase inhibitor, ephrin-A5 failed to inhibit ERK phosphorylation. Quantification depicts P-ERK levels relative to control (without treatment and inhibitor set to 1) for OA and SV. (D) Ephrin-A5 does reduce BDNF-stimulated P-ERK levels in the presence of phosphatase inhibitors.

P1 hippocampal or E17.5 cortical cultures were incubated in NMEM/B27 medium as described previously [36]. In brief, neurons (5×10^3^–10^4^) were cultured on poly-L-lysine (100 µg/ml; Sigma) and laminin (20 µg/ml; Gibco) coated coverslips (13 mm). Ephrin-A5-Fc (R&D systems) or Fc alone (Sigma) was applied to the culture medium at 1 µg/ml, both pre-clustered with 10 µg/ml anti-human IgG Fc-specific (Sigma) for 30 mins at 37°C. BDNF was applied at 10 ng/ml (Fig. 1). Stimulation was performed for 45 min at 37°C (Fig. 1). For experiments with U-0126 (Cell Signaling), cells were pre-incubated with U-0126 at 10 µM for 1h before application of BDNF. For biochemistry (Fig. 2) and qRT-PCR, cortical neurons (approx. 10^6^ cells/35 mm dish coated with poly-L-lysine at 10 µg/ml) were cultured for 3 div (days *in vitro*) prior to stimulation (see below). We used nucleofection (Amaxa, Cologne, Germany) to deliver a constitutively-active mutant of MEK1 [44] into cortical or hippocampal neurons.

### Biochemistry

Cortical cultures were stimulated with 1 µg/ml clustered (see above) ephrin-A5-Fc and/or BDNF (10 ng/ml) in NMEM/B27 medium for the indicated time periods. Phosphatase inhibitors were applied for 15 mins prior to ephrin-A5-Fc and/or BDNF stimulation: sodium vanadate (200 µM; Sigma), okadaic acid (200 nM; Calbiochem), PhosStop (1x; Roche). Cells were lysed in 100 mM Tris pH 7.8, 150 mM NaCl, 1 mM EDTA, 1% Triton-X-100, 0.1% SDS and protease inhibitors (Roche). Samples were resolved on 10-12% SDS-PAGE, followed by transfer on PVDF membranes (Amersham). After blocking, first antibodies were applied overnight at 4°C: rabbit anti-ERK (1∶1000; Cell Signaling), rabbit anti-P-ERK (1∶1000; Cell Signaling), mouse anti-GAPDH (1∶50000), rabbit anti-TrkB (1∶1000; Santa Cruz), rabbit anti-P-TrkB (1∶1000; Cell Signaling), mouse anti-EphA4 (1∶750; BD Transduction Laboratories), P-EphA3 (1∶10000, gift of Dr. M. Greenberg, Harvard Medical School, Boston). Detection of first antibodies involved horseradish-peroxidase conjugated secondary antibodies (1∶5000) and the ECL Western Blotting Substrate (Pierce).

### Quantitative real-time PCR (qRT-PCR)

Cultures were stimulated with 2.5 ng/ml BDNF and/or 1 µg/ml pre-clustered ephrin-A5-Fc (see above). Total RNA was isolated with the RNeasy kit (Qiagen). Reverse transcription was performed with 1 µg RNA using reverse transcriptase (Promega) and random hexamers. qRT-PCR was performed on ABI PRISM 7700 Sequence Detector with the Power PCR SYBR green PCR master mix (Applied Biosystems). Expression was determined in relation to *Gapdh* RNA levels. Primer sequences can be obtained upon request.

### Immunocytochemistry

Cells were fixed for 15 minutes in 4% PFA/5% Sucrose/PBS, permeabilized for 5 minutes in 0.1% Triton-X-100/PBS and blocked for 30 minutes in 2% BSA/PBS. Primary antibodies were incubated overnight at 4°C as follows: mouse α-ß-tubulin (1∶5000; Sigma), mouse α-class III ß-tubulin (1∶1000; Covance), rabbit anti-ERK (1∶300; Cell Signaling), rabbit anti-P-ERK (1∶300; Cell Signaling). First antibodies were detected with Alexa 488 or 546-conjugated secondary antibodies (1∶1000; Molecular Probes). Cells were stained for F-actin with Texas Red-X Phalloidin (1∶100; Molecular Probes).

For visualization of P-ERK and Eph receptors, cultures were incubated with 1 µg/ml pre-clustered ephrin-A5 for 20 minutes, followed by fixation with 4%PFA/sucrose for 15 minutes. Ephrin-A5 bound to Eph receptors was visualized with anti-Fc Cy3 conjugated antibodies (1∶300 in block). Subsequently, P-ERK staining was performed as indicated above.

### Image acquisition and statistics

Pictures were acquired on a Zeiss Axiovert 200M or Zeiss LSM confocal microscope using an Axiocam camera and Axiovision software. We used 10x, 20x and 63x Zeiss objective lenses with numerical apertures of 0.3, 0.8 and 1.3 (oil), respectively. Pictures were further processed using Photoshop software (Adobe).

**Figure 3:**
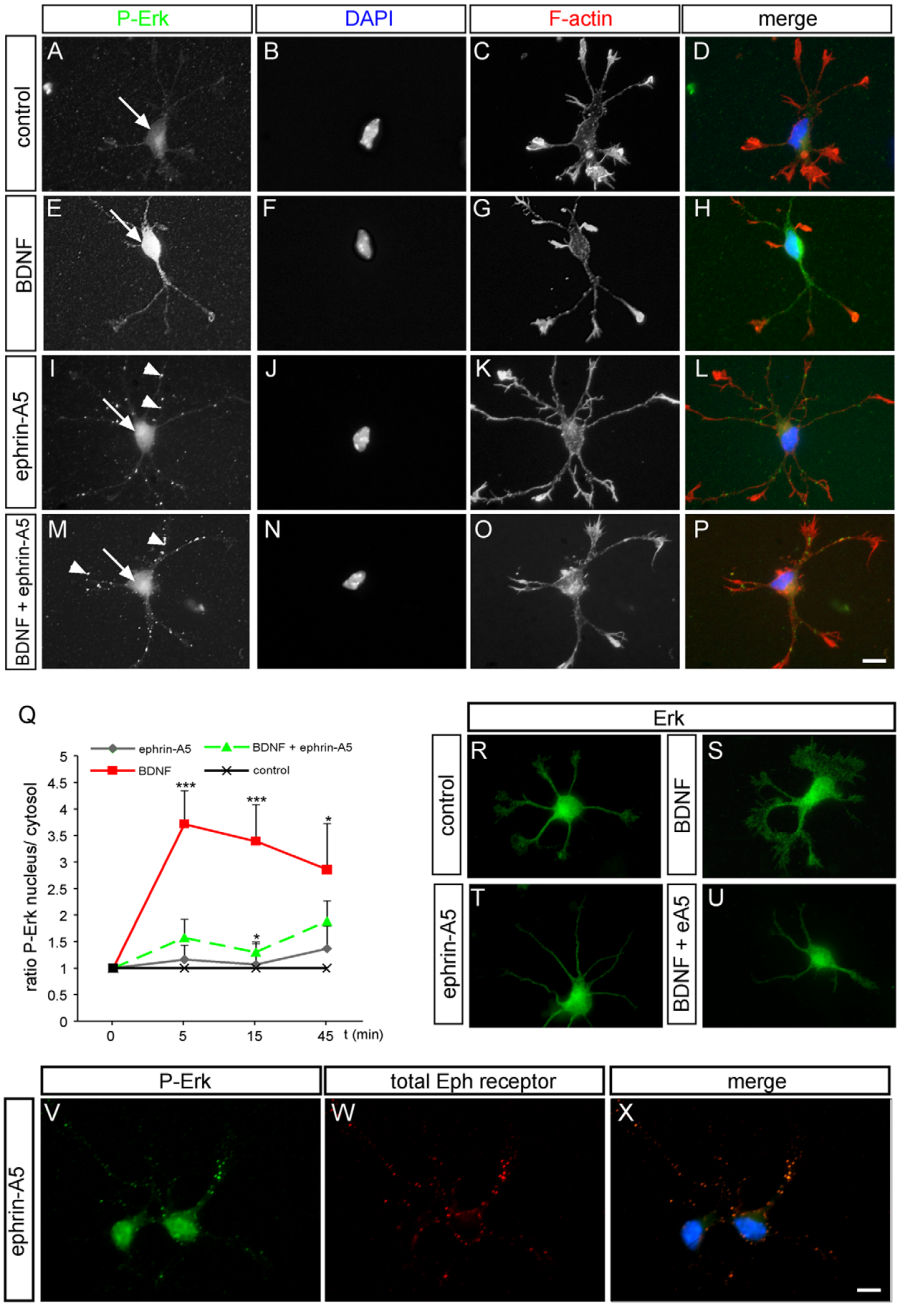
Eph forward signaling prevents nuclear ERK translocation. Wild-type hippocampal neurons were treated with guidance cues as indicated, followed by staining for P-ERK (green), DAPI (blue) and F-actin (red). Arrows point at the nucleus. (A-D) In untreated neurons, nuclear P-ERK levels were low. (E–H) BDNF enhanced P-ERK levels, which accumulated in and around the nucleus. (I–L) Ephrin-A5 did not increase nuclear P-ERK accumulation. However, P-ERK positive clusters were observed in the neurite (arrowhead in I). (M-P) Ephrin-A5, when applied along with BDNF, antagonized nuclear P-ERK entry evoked by BDNF. As with ephrin-A5 (I), P-ERK localized to neurites (arrowheads in M). (Q) Quantification of nuclear P-ERK. The ratio of P-ERK in the nucleus vs. cytosol was plotted against the three time-points of stimulation. Values of ratios for untreated neurons were set to 1. Statistical significance is provided relative to control (untreated) values. (R-U) Total ERK levels remained constant throughout the various treatments. (V) Hippocampal neurons stained for P-ERK localization. Ephrin-A5 stimulation induces clusters of P-ERK in neurites. (W) All Eph receptors bound by ephrin-A5 were labeled. Typically, Eph receptors were found in clusters localized to the neurites. (X) A merged image of (V) and (W), revealing co-localization of P-ERK and Eph receptors in hippocampal neurites. Scale-bar (A-P, R-X)  =  10 µm.

For data in Fig. 1 and Fig. S1, four independent experiments were performed. In each experiment 20 neurons/condition were analyzed. Filopodia number (>1 µm) and growth cone surface was determined for all growth cones/neuron using Axiovision software. For quantification of neurite number and branches, only neurites of more than 30 µm and branches of more than 2 µm were included. In Fig. 2, three independent cultures/condition were analyzed using Image Quant software (Molecular Dynamics). To evaluate ERK nuclear translocation (Fig. 3), four independent experiments including 25 neurons/experiment/condition were analyzed. A ratio of cytoplasmic vs. nuclear P-ERK intensity was determined using Axiovision software. In qRT-PCR, RNA of at least three independent cultures/condition was harvested.

Statistical significance was assessed by using a two-tailed t-test (Excel software) with *, **, *** representing *P*≤0.05, 0.01, 0.001. Error bars indicate standard deviation (s.d.) if not indicated otherwise.

## Results

### Ephrin-A5 stimulation counteracts BDNF-induced neuronal motility in vitro in an SRF-dependent manner

To study an interplay between ephrin-A and BDNF stimulated neuronal signaling, we investigated growth cone morphology (Fig. 1) and neurite branching (Fig. S1) of primary hippocampal neurons. In a first set of experiments, neurons were incubated with soluble ephrin-A5, BDNF or both together for 45 minutes (Fig. 1). In line with retinal growth cones [45], BDNF enhanced filopodia number, length and overall growth cone area (Fig. 1E, F) compared to non-treated wild-type neurons (Fig. 1A, B; see quantification in Q and R). Contrastingly, ephrin-A5 resulted in growth cone collapse as reported before [36], [37], [46]. When simultaneously adding ephrin-A5 and BDNF, growth cones collapsed (Fig. 1M, N). A similar result was recently reported for retinal growth cones [47]. Besides filopodia number (Fig. 1Q) and overall growth cone area (Fig. 1R), ephrin-A5 also counteracted a BDNF-mediated increase in filopodia length (data not shown).

Next, we asked whether both guidance cues require SRF gene activity to influence growth cone motility. As before [36], [37], *Srf* mutant growth cones lacked filopodia, therefore appearing in a “rounded” shape (Fig. 1C, D). Neuronal phenotypes evoked by SRF deficiency, including reduced growth cone filopodia number, decreased branching and neurite outgrowth can be rescued by expression of constitutively-active SRF-VP16 in SRF deficient neurons [36], [48]. BDNF failed to induce filopodia (Fig. 1G, H), ephrin-A5, alone (Fig. 1K, L) or together with BDNF (Fig. 1O, P), was unable to induce a full growth cone collapse in *Srf* mutant neurons. Instead, ephrin-A5 alone (Fig. 1K, L [36], [37] or together with BDNF (Fig. 1O, P) induced F-actin and microtubule rings in SRF deficient growth cones.

Besides growth cone morphology (Fig. 1), we analyzed neurite and secondary branch formation (Fig. S1). BDNF stimulated neurite and branch formation was inhibited by ephrin-A5 in an SRF dependent manner (Fig. S1). This BDNF function most likely requires MAP kinase signaling as shown in many instances before [25], [26], [27]. In accordance, BDNF mediated short-term stimulation to increase filopodia number and growth cone area (Fig. 1S, T) required MAP kinase signaling as demonstrated by pharmacological interference with U-0126, a MEK inhibitor. Further, long-term effects stimulated via BDNF such as an increase in primary neurite number (Fig. S1) was likewise dependent on ERK signaling.

In sum, Eph forward signaling impaired BDNF-induced growth cone motility and neurite/branch formation *in vitro*. Similar to NGF-mediated responses on peripheral axons [30], BDNF signaling required SRF mediated gene expression.

### Ephrin-As counteract BDNF signaling via suppression of ERK kinase activity

Next, we investigated the underlying signaling mechanism, whereby ephrin-As and neurotrophins might interact. Focus was given on MAP kinases and especially ERK as a possible signaling mediator regulated by receptors of both guidance cues (see Introduction). Upon activation by ephrins, Eph receptors activate [49], [50], [51] or inactivate [46], [52], [53], [54], [55], [56] MAP kinases depending on e.g. cell type and Eph subclass implicated.

For these biochemical experiments requiring larger amounts of neuronal tissue, cortical neurons rather than hippocampal neurons were employed (Fig. 2). Both neuronal cell types behave identical in many parameters (ephrin-A5 induced growth cone collapse, ERK activation and e.g. BDNF-evoked gene expression; own data not shown). Therefore, data obtained should be interchangeable with both neuron types.

Short-term activation of Eph forward signaling by ephrin-A5 application suppressed ERK kinase activation as revealed by decreased ERK1 and ERK2 phosphorylation (P-ERK). In agreement with our data, EphA activation also decreased ERK signaling in hippocampal neurons [46] and cortical neurons [55]. During longer exposure to ephrin-A5 (>1h), P-ERK returned to control levels (Fig. 2A).

Application of BDNF alone expectedly increased P-ERK levels (Fig. 2B). Notably, when ephrin-A5 was co-applied with BDNF, ERK activity was reduced. This suggests that ephrin-A5 counteracted ERK activation by BDNF (Fig. 2B).

One way by which ephrin-A5 might inhibit ERK phosphorylation is recruitment of phosphatase activity, which has been implicated as downstream effector of an EphA mediated growth cone collapse [46]. Indeed, pre-incubation with various phosphatase inhibitors (i.e. okadaic acid, OA, blocking serine/threonine phosphatases; sodium vanadate, SV, a protein-tyrosine phosphatase inhibitor and a pan-phosphatase inhibitor coctail, mix) prevented ephrin-A5 from inhibiting ERK activity (Fig. 2C). EphA receptor phosphorylation assessed by P-EphA3 levels was not obviously altered by application of either phosphatase inhibitor (Fig. 2C and data not shown). We further analyzed whether ephrin-A5 also requires phosphatases to reduce BDNF-induced P-ERK levels (Fig. 2D; n = 3 independent experiments). In contrast to ephrin-A5 alone (Fig. 2C) we did not observe an major requirement of phosphatases to mediate this effect of ephrin-A5 on BDNF-mediated ERK phosphorylation (Fig. 2D). Only in the presence of the phosphatase inhibitor mix (mix) we observed a slight reconstitution of P-ERK levels comparable to BDNF treatment alone (Fig. 2D).

### Eph forward signaling induces ERK activation within the neurites while impairs BDNF induced p-ERK activation and its subsequent nuclear translocation

Subsequently to ERK phosphorylation, neurotrophins stimulate cytoplasmic to nuclear P-ERK translocation allowing for modulation of neuronal gene expression [24], [57]. Thus, in addition to biochemical experiments (Fig. 2), immunofluorescence microscopy was employed to investigate the sub-cellular localization of P-ERK in neurons treated under the various conditions (Fig. 3). Indeed, stimulation of neurons for 15 to 45 minutes with BDNF alone (Fig. 3E-H) expectedly elevated nuclear P-ERK levels (indicated by arrows) compared to non-treated cells (Fig. 3A-D; see quantification in Q). This finding is in line with Western Blotting experiments (Fig. 2B) showing increased P-ERK levels upon BDNF treatment. Next, we analyzed whether ephrin-A5 blocks BDNF signal propagation on the level of nuclear P-ERK accumulation (Fig. 3). Eph receptor activation by ephrin-A5 prevented ERK activation as measured by P-ERK levels in the nucleus, (Fig. 3I-L and Q). Notably, upon ephrin-A5 application P-ERK now accumulated in neurites (arrowheads Fig. 3I). Similar to incubation with ephrin-A5 alone, we observed that ephrin-A5 co-applied with BDNF was able to reduce nuclear P-ERK (Fig. 3M-P). Instead, as seen for ephrin-A5 alone (Fig. 3I), we observed P-ERK signals in neurites (arrowhead Fig. 3M). Total ERK levels were not obviously altered by any of the treatments (Fig. 3R-U; see also Fig. 2). Of note, these P-ERK clusters co-localized with Eph receptors in neurites upon ephrin-A5 application (Fig. 3V-X) but not in the un-stimulated situation (data not shown).

In sum, Eph forward signaling impaired BDNF mediated P-ERK activation.

### Eph forward signaling modulates BDNF-evoked IEG expression

**Figure 4:**
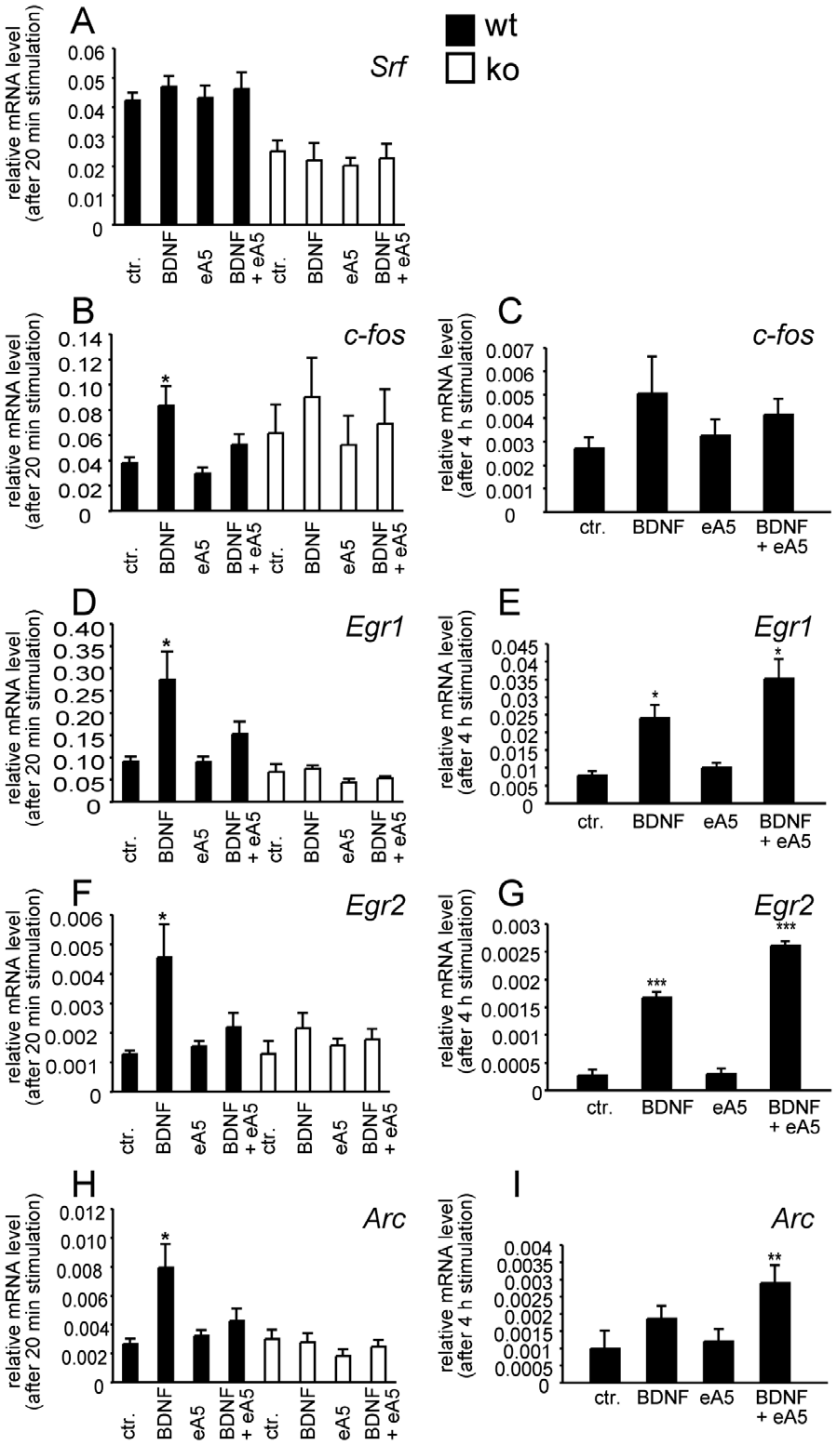
The BDNF-evoked neuronal IEG response is counteracted by ephrin-A5 co-application. Wild-type (black bars) and *Srf* mutant (white bars) cortical neurons were treated for 20 minutes (A, B, D, F, H) or 4h (C, E, G, I) with guidance cues, followed by quantification of mRNA levels of indicated genes. (A) *Srf* mRNA levels were not altered by any treatment. In conditional *Srf* mutant cultures, *Srf* mRNA levels are reduced approximately two-fold compared to wild-type. (B, D, F, H) Expression of the IEGs *c-fos* (B), *Egr1* (D), *Egr2* (F) and *Arc* (H) was up-regulated in wild-type neurons by BDNF, but not ephrin-A5 alone. Ephrin-A5 inhibited BDNF-evoked IEG up-regulation in wild-type neurons. As revealed by *Srf* mutant neurons, BDNF-induced gene regulation of *Egr1*, *Egr2*, *Arc* and weaker *c-fos* required SRF. (C, E, G, I) Long-term exposure to BDNF, but not ephrin-A5 alone, elevated expression of the IEGs *c-fos* (C), *Egr1* (E), *Egr2* (G) and *Arc* (I) in wild-type neurons. At this extended exposure (4h) to both guidance cues, co-application of ephrin-A5 failed to decrease BDNF-stimulated gene expression. This is inline with the activation profile of Eph receptors reaching their maximum at 10-60 mins after ephrin-A5 addition (see Fig. 2).

Nuclear P-ERK enhances gene transcription by activating many transcription factors, including SRF [31]. As Ephrin-A5 impairs BDNF elicited ERK activation and its subsequent nuclear translocation, we addressed the potential consequences of Ephrin-A5 treatment on BDNF-mediated gene expression (Fig. 4). Focus was given on a well-established neurotrophin mediated gene expression program, the IEG response (see introduction). For this, cortical neurons derived from wild-type or *Srf* mutant embryos were treated for 20 minutes or 4h with ephrin-A5, BDNF or both substances applied simultaneously. Subsequently, IEG mRNA levels were assessed by quantitative real-time PCR.

BDNF alone elicited in wild-type neurons a robust IEG response at both time-points, as revealed by induction of *c-fos* (Fig. 4B, C), *Egr1* (Fig. 4D, E), *Egr2* (Fig. 4F, G) and *Arc* (Fig. 4H, I). *Srf* abundance was not altered by this short BDNF exposure (i.e. 20 mins; Fig. 4A) in contrast to longer exposure times [32]. Eph forward signaling alone did not obviously alter IEG induction at any time-point (Fig. 4A-I). Of note, when BDNF and ephrin-A5 were applied together, short-term activation (i.e. 20 min) of Eph forward signaling counteracted BDNF-evoked induction of the IEGs *c-fos*, *Egr1*, *Egr2* and *Arc* (Fig. 4B, D, F, H). Now, mRNA abundance of IEGs almost returned to control level. This counteraction of BDNF-mediated IEG responses by ephrin-A5 was not seen at a longer ephrin-A5 exposure time (i.e. 4h; Fig. 4C, E, G, I). This observation is in line with EphA forward signaling reaching its maximum intensity (as assessed by P-EphA3) between 10 - 60 mins of ephrin-A5 exposure and decreasing at longer incubation times (see Fig. 2A).

We next investigated whether modulation of a BDNF-mediated IEG induction by ephrin-A5 is a general feature of repulsive guidance cues. Short-term addition (20 min) of Semaphorin 3A, an established growth cone collapsing agent [58], was unable to repress BDNF-evoked IEG responses (Fig. S2). This result indicates a rather specific property of ephrin-As on neurotrophin signaling.

Finally, we asked whether the gene regulator SRF is required for these short-term neurotrophin-mediated IEG responses (Fig. 4B, D, F, H). Our data, employing *Srf* mutant neurons, suggest that the BDNF induced IEG response requires SRF. This is supported by a reduced induction of the IEGs *Egr1, Egr2, Arc* (Fig. 4D, F, H) and to a lesser extent *c-fos* (Fig. 4B) in *Srf* mutant neurons.

Taken together, Eph forward signaling modulates neurotrophin mediated IEG induction.

### ERK signaling is involved in decreasing BDNF-induced gene activity via Eph receptors

**Figure 5:**
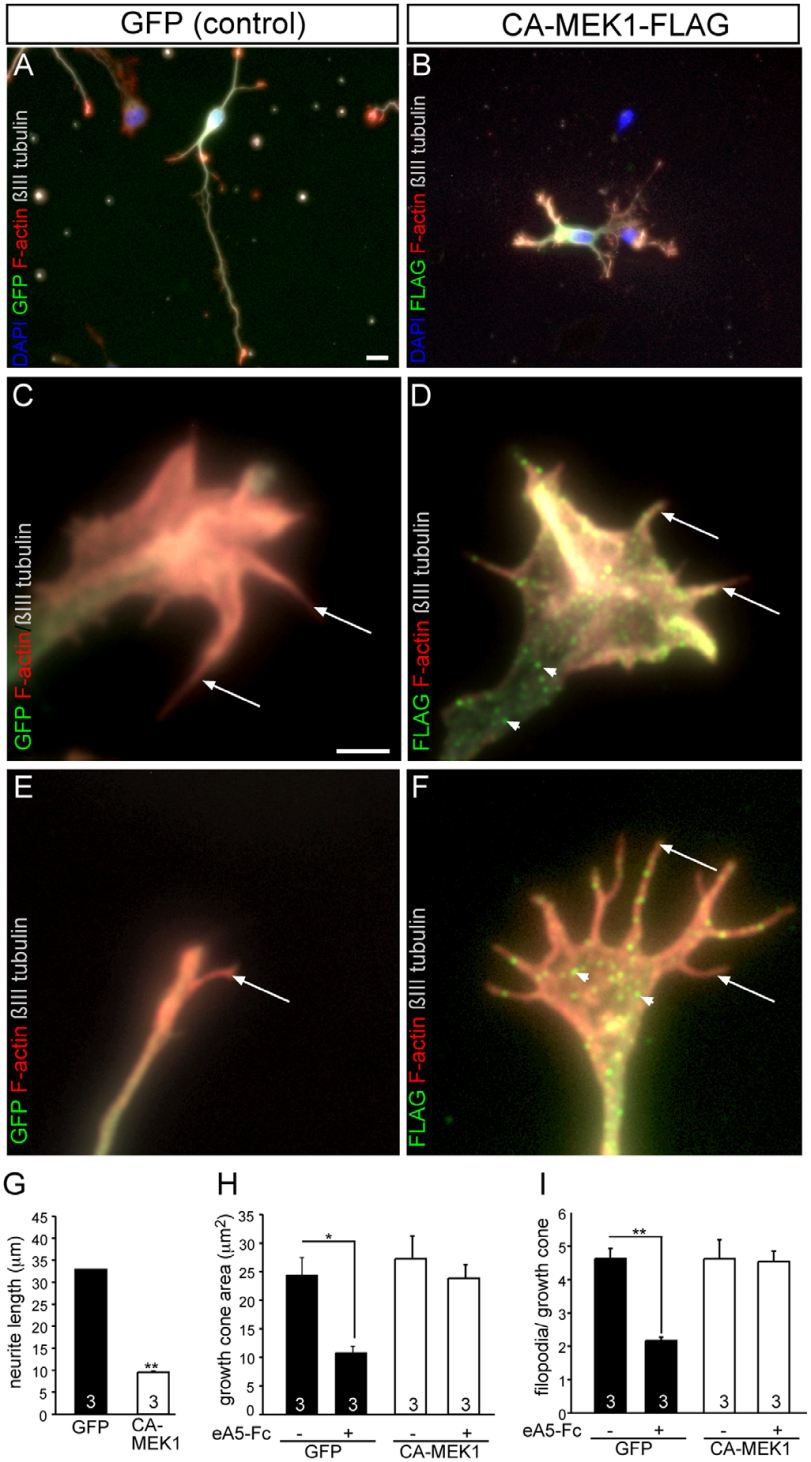
Constitutively-active MEK1 blocks ephrin-A5 mediated growth cone collapse. Wild-type neurons were electroporated with vectors expressing GFP (control; A, C, E) or expressing a FLAG-tagged constitutively-active MEK1 (CA-MEK1; B, D, F). Neurons were treated with ephrin-A5-Fc for 30 min followed by visualization of GFP (A, C, E) or CA-MEK-1 (green in B, D, F), F-actin and ßIII tubulin. (A, B) Overexpression of CA-MEK1 (B) reduces mean neurite length compared to a control GFP-expressing neuron (A). (C-F) A control growth cone without ephrin-A5 application (C) typically protrudes multiple filopodia (arrow). CA-MEK-1 expressing growth cones (D) were indistinguishable from a GFP-expressing growth cone. Note that CA-MEK1 was expressed in a dot-like pattern in the growth cone (arrowheads in D and F). Upon ephrin-A5 application, a control growth cone (E) collapsed resulting in only few filopodia. In contrast, a CA-MEK1 expressing growth cone (F) was only partially collapsed and many filopodia structures were preserved after ephrin-A5 induction. (G) Quantification of average neurite length. (H, I) Quantification of growth cone area (H) and filopodia number/growth cone (I) in the four conditions. Ephrin-A5 induces a growth cone collapse, resulting in reduced growth cone area and filopodia number in GFP but not CA-MEK1 expressing neurons. Scale-bar (A, B)  =  10 µm; (C-F)  =  2 µm.

**Figure 6:**
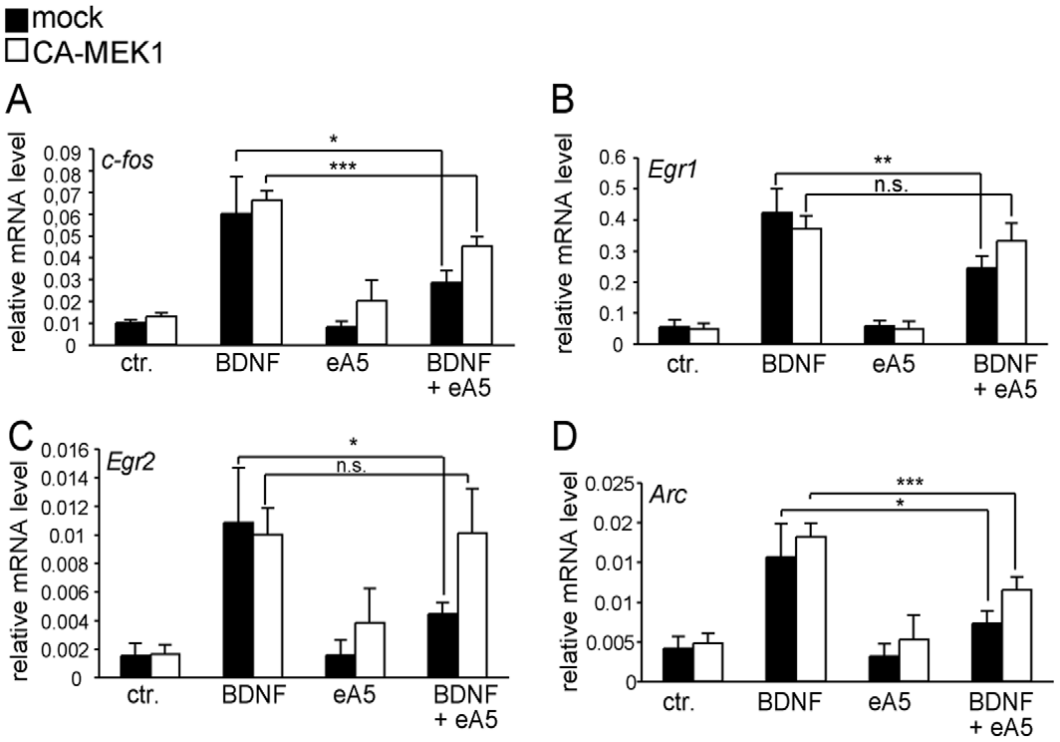
Ephrin-A5 suppresses BDNF-induced IEG responses via MAP kinase signaling. Wild-type cortical neurons, mock-electroporated (black bars) or with a vector expressing constitutively-active MEK1 (white bars), were treated with guidance cues for 20 minutes as depicted, followed by mRNA quantification. In mock-electroporated neurons, BDNF induced an IEG response of *c-fos* (A), *Egr1* (B), *Egr2* (C) and *Arc* (D). Co-application of ephrin-A5 and BDNF reduced mRNA levels of all four IEGs in mock-electroporated neurons (A-D). In contrast, expression of constitutively-active MEK1 prevented Eph forward signaling from suppressing BDNF-evoked IEG responses. This was most evident for the IEGs *Egr1* (B) and *Egr2* (C).

Data provided so far suggest that ephrin-A5 might counteract BDNF-mediated gene expression and growth cone responses via decreasing ERK activity and nuclear localization (Figs. 2 and 3). To test this hypothesis more directly, we analyzed whether Eph forward signaling can still suppress BDNF-stimulated IEG induction and growth cone collapse when ERK kinase activity was experimentally raised. For this, ERK activity was elevated by expressing constitutively-active MEK1 (CA-MEK1), an ERK upstream activator, in cortical neurons (Fig. S3). Subsequently, neurons were subjected to either an ephrin-A5 mediated growth cone collapse assay (Fig. 5) or stimulated with guidance cues for 20 minutes and IEG mRNA levels were quantified via qRT-PCR (Fig. 6).

First of all, we analyzed the impact of CA-MEK1 on neurite outgrowth and ephrin-A5 mediated growth cone responses (Fig. 5). The average neurite length of neurons overexpressing CA-MEK1 (Fig. 5B) was clearly reduced compared to control GFP expressing neurons (Fig. 5A; see quantification in (G)). Growth cones of GFP (C) and CA-MEK1 expressing neurons were similar and protruded many filopodia (arrows in Fig. 5C-F). CA-MEK1 expression in growth cones was confined to individual dot-like structures (arrowheads in Fig. 5D, F). Ephrin-A5 application on a GFP expressing control growth cone (Fig. 5E) resulted in a growth cone collapse as revealed by reduced overall growth cone area and filopodia number (quantified in Fig. 5H, I). In contrast, in neurons overexpressing CA-MEK1 (Fig. 5F), ephrin-A5 induced only a partial growth cone collapse. Thus, growth cone area and filopodia number were not reduced (see Fig. 5H, I). This result suggests that an ephrin-A5 induced signaling cascade targets MAP kinase signaling to exert a full growth cone collapse. This result is in contrast to a recent report [46] which excluded ERK signaling downstream of an ephrin-A5 mediated growth cone collapse.

Next, we tested whether ephrin-A5 signaling suppresses ERK kinase signaling also to interfere with BDNF-mediated gene expression (Fig. 6). In agreement with previous results (Fig. 4), in mock-electroporated neurons, ephrin-A5 incubation reduced the BDNF-mediated up-regulation of *c-fos* (Fig. 6A), *Egr1* (Fig. 6B), *Egr2* (Fig. 6C) and *Arc* (Fig. 6D) mRNA. In contrast, in neurons over-expressing CA-MEK1, ephrin-A5 could not suppress BDNF-induced IEG responses to the same degree. Particularly *Egr1* (Fig. 6B) and *Egr2* (Fig. 6C) mRNA levels were almost identical when comparing neurons treated with BDNF alone and neurons with BDNF and ephrin-A5 together.

Thus, ephrin-A5 represses gene activity elicited by BDNF at least in part via MAP kinases.

Besides IEGs, we inspected the influence of both guidance cues on cytoskeletal genes, whose gene products might modulate cytoskeletal dynamics evoked by ephrin-As and/or neurotrophins (Fig. S4). Therefore, the filamentous actin (F-actin) stabilizing tropomyosins (*Tpm1* and *Tpm2*), the F-actin cross-linker alpha-actinin 1 (*Actn1*) and the motor protein dynein light chain 1 (*Dnal1*) were analyzed. mRNA levels of other cytoskeletal genes were not altered by any of the guidance cues (i.e. cofilin, filamin A, mena, vinculin and smooth muscle actin; data not shown). Short-term stimulation with guidance cues did not result in major alterations of cytoskeletal mRNA levels except for upregulation of *Tpm1* by ephrin-A5 (Fig. S4A). At 16h of incubation, both tropomyosin genes were induced by BDNF and ephrin-A5 alone and both together (Fig. S4B, D). *Actn1* levels were induced by individual application of ephrin-A5 and BDNF and by co-application of both (Fig. S4F). A similar profile was observed for the dynein light chain (Fig. S4H).

Taken together, cytoskeletal gene expression was modulated by ephrin-A5 and BDNF. In contrast to the antagonistic impact on the IEG gene response (Figs. 4 and 6), ephrin-A5 and BDNF resulted in rather synergistic action on cytoskeletal gene expression. This difference might be due to short-term (i.e. 20 minutes) vs. long-term (i.e. 16h) exposure of both guidance cues in the IEG and cytoskeletal gene response, respectively.

### EphA7 modulates growth cone morphology yet is dispensable for ephrin-neurotrophin communication

**Figure 7:**
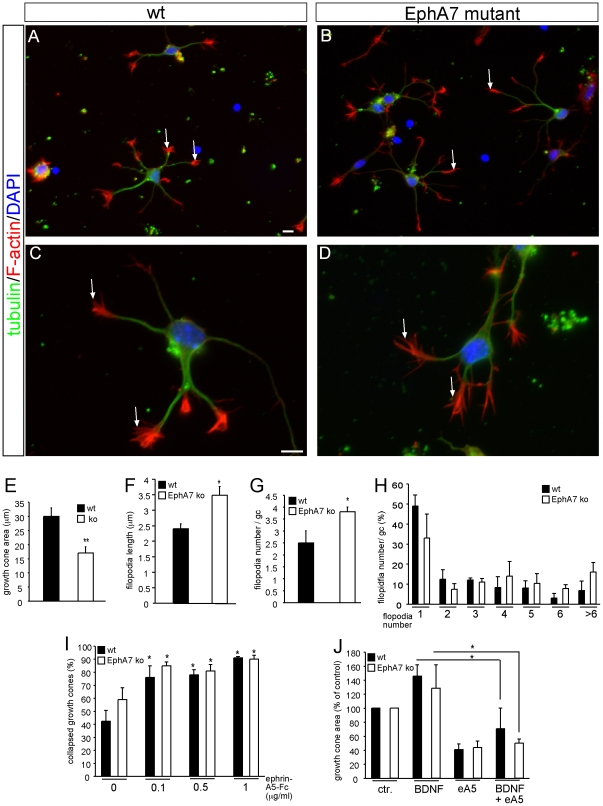
EphA7 modulates growth cone morphology yet is dispensable for ephrin-neurotrophin communication. Hippocampal neurons derived from wild-type (A, C) and EphA7 deficient mice (B, D) were stained for DAPI (blue), tubulin (green) and F-actin (red) expression. Individual growth cones in (A–D) are labeled by an arrow. (A–D) The area of individual growth cones of wild-type neurons (A; higher magnification in C) exceeds that of EphA7 deficient growth cones (B; higher magnification in D). Filopodia length and number was elevated in EphA7 deficient growth cones compared to control. (E) Quantification of growth cone area. *Epha7* mutant growth cone area was reduced almost two-fold compared to wild-type. (F) The length of individual filopodia is increased in EphA7 deficient growth cones compared to wild-type. (G, H) The total number of filopodia/growth cone is increased in EphA7 deficient growth cones compared to control (G). (H) depicts the distribution (in percentage) of growth cones harboring a certain filopodia number (1-6 and >6). The frequency of growth cones with 6 or more filopodia is almost doubled upon EphA7 deletion. (I) EphA7 is dispensable for transducing ephrin-A5 collapsing activity in hippocampal neurons. Addition of three concentrations of ephrin-A5 resulted in a comparable growth cone collapse induction in wild-type and EphA7 deficient neurons. (J) EphA7 is not required for ephrin-A mediated suppression of BDNF-evoked growth cone motility. In the absence of EphA7, ephrin-A5 and BDNF co-administration reduced the growth cone area (J), filopodia length and number (data not shown) elevated by BDNF alone. Scale-bar (A, B)  =  10 µm; (C, D)  =  5 µm.

In order to uncover a specific Eph receptor family member (EphA1-EphA8, EphB2) transducing ephrin-A5's impact on neurotrophin signaling, EphA7 was further analyzed (Fig. 7). EphA7 is expressed on hippocampal neurons (e.g. [59]) and EphA7 ablation results in aberrant axon guidance processes [15]. Therefore EphA7 might be potential candidate receptor for mediating ephrin-A5's influence on neurotrophin signaling in hippocampal neurons.

Hippocampal neurons derived from wild-type or EphA7 deficient mice were indistinguishable with regard to overall neurite length (data not shown). In contrast, growth cone morphology of *Epha7* mutant neurons (arrows Fig. 7B, D) was altered compared to wild-type (arrows Fig. 7A, C). EphA7 deficient cultures were readily distinguishable from control cultures by the reduced overall area taken up by individual growth cones (Fig. 7E). Although overall growth cone area was reduced (Fig. 7E), filopodia length and number were increased upon EphA7 deletion (Fig. 7F–H).

Next, sensitivity of *Epha7* mutant neurons towards ephrin-A5 was tested in the growth cone collapse assay (Fig. 7I). Employing three different ephrin-A5 concentrations, we observed a full growth cone collapse response for both wild-type and EphA7 deficient neurons. This result suggests that EphA7 is not a crucial EphA receptor on hippocampal neurons for mediating ephrin-As repulsive guidance activity. Also, due to the promiscuity in Eph-ephrin interactions (see introduction) other Eph receptors expressed on hippocampal growth cones (e.g. EphA4, EphA5, EphA6, EphB2) might transduce ephrin-A5's collapsing activity.

Finally we tested whether ephrin-A5 requires EphA7 to repress neurotrophin mediated neuronal motility (Fig. 7J). Similar to wild-type neurons, we observed that co-application of ephrin-A5 and BDNF in EphA7 deficient growth cones still resulted in a decrease of the growth cone area (Fig. 7J) as well as filopodia length and number (data not shown). This suggests that EphA7 is not required to transmit ephrin-A5's impact on neurotrophin signaling. As above, other Eph receptors are likely compensating for EphA7 deficiency or fulfill the major ephrin-A5 receptor function to interact with neurotrophin signaling.

In sum, EphA7 modulates growth cone morphology yet is dispensable as ephrin-A5 receptor to suppress neurotrophin signaling.

## Discussion

In this study, we explored consequences of primary neurons stimulated simultaneously with two guidance cues, ephrin-A5 and BDNF. BDNF application on primary hippocampal neurons resulted in enhanced growth cone dynamics and branch formation (Fig. 1, Fig S1). These findings are in line with the “attractive” guidance properties reported for BDNF [16], [19], [21], [23], [60]. Eph forward signaling counteracted filopodial dynamics and neurite formation/branching induced by BDNF (Fig. 1 and Fig. S1). How might these findings translate to axon guidance processes in the hippocampus *in vivo*? Similar to axonal pruning in the visual system [16], [19], BDNF might promote the initial overshooting of hippocampal axons by stimulating branching. Subsequently, ephrin-A5 might via EphA and potentially also EphB2 forward signaling trigger axonal retraction, e.g. pruning of hippocampal infrapyramidal mossy fiber axons. In line with this model is a reported function of Eph family members in hippocampal pruning *in vivo* [5], [7]. In addition to Eph forward signaling, ephrin-A reverse signaling is likewise suppressing BDNF-mediated branch formation [16]. Thus, both reverse and forward signaling by Eph family members counteracts BDNF activity. The Eph/BDNF interaction has been recently shown to be not only important for processes of axon guidance but also synapse function [61].

How and where do ephrin-A and BDNF signaling interact? We have not observed co-localization of EphA and TrkB receptors (data not shown) and individual receptor activation was not impaired by BDNF and ephrin-A5 co-application (Fig. 2). This argues against direct interaction at the level of the Eph and TrkB receptor and points at an interaction taking place on converging intracellular signaling cascades as observed for the EphA and Ret interaction before [42], [62]. Indeed, data provided in this study suggest that ephrin-A5 induced Eph forward and a BDNF induced signaling cascade converge on ERK signaling. In line with others [46], [55], ephrin-A5 alone suppressed ERK phosphorylation. This downregulation of P-ERK by ephrin-A5 appears to involve phosphatases (Fig. 2C). In addition, we here show that ephrin-A5 counteracted BDNF-induced ERK activity and reduced therefore P-ERK levels in the nucleus (Figs. 2 and 3). Here, ephrin-A5 does not obviously dependent on phosphatases to reduceP-ERK levels induced by BDNF (Fig. 2D).Notably, ephrin-A5 application resulted in P-ERK signals in the neurites, at positions co-localizing with Eph receptors (Fig. 3). Thus, upon activation of Eph forward signaling, P-ERK might be sequestered in neurites and thereby precluded from nuclear transport. This, subsequently would diminish the nuclear P-ERK pool and reduce the activity of ERK-responding gene regulators such as SRF. Currently, the mechanism by which Eph receptors might sequester P-ERK in neurites is unresolved. This mechanism might involve direct complex formation of Eph receptors and P-ERK (Fig. 3V-X). Hence, in growth cones simultaneously activated by multiple guidance cues, one function of Eph mediated P-ERK clustering in neurites might be the modulation of the signaling strength of other guidance cues as demonstrated for neurotrophins in this study.

Signaling by many receptor tyrosine kinases modulates gene activity. Contrastingly, Eph receptor signaling has so far not been unambiguously associated with gene transcription in neurons. We demonstrated that ephrin-A5 can suppress BDNF-mediated gene expression (Fig. 4). This, at least in part, appears to be mediated via the potential of ephrin-A5 stimulated signaling to inhibit ERK kinases (Figs. 5 and 6). How might ephrin-A5, via counteracting a BDNF-mediated IEG response, affect neuronal motility? IEGs such as *c-fos* and *Egr1* can stimulate neuronal motility [63], [64]. Thus, BDNF-mediated up-regulation of these IEGs might contribute to BDNF's neurite- and branch promoting activity [16], [21]. In such a model, Eph receptor signaling might inhibit BDNF-induced neurite branching by lowering IEG levels. In addition to *c-fos* and *Egr-1* emerges *Arc* as an interesting IEG relevant to neuronal motility and synapse formation [65], [66]. Arc regulates actin cytoskeletal dynamics by inhibiting the actin-severing protein cofilin. In addition to IEG modulation, we observed that long-term ephrin-A5 exposure modulates gene expression of actin cytoskeletal genes such as tropomyosins and actinin (Fig. S3). This might be a first hint that Eph forward signaling modulates neuronal motility also via induction of gene activity.

Induction of all IEGs required the transcription factor SRF (Fig. 4). So far, CREB (cyclic AMP response element binding protein) is considered as a major gene regulator underlying neurotrophin-induced gene expression [29]. Our data, together with others [30], [32], suggest that in addition to CREB, SRF emerges as transcription factor activated by neurotrophins. In primary neurons investigated in this study, CREB was unable to compensate for SRF-deficiency (Fig. 4). This study did not resolve whether gene products transcribed by SRF upon activation by BDNF are subsequently anterogradely transported and are required to mediate BDNF's impact on growth cone function. It also should be noted that actin cytoskeletal dynamics in growth cones of SRF-deficient neurons might be generally impaired in such way that many guidance cues including ephrins, BDNF and semaphorins might not be able to modulate growth cones according to their usual outcome (e.g. collapse or filopodia extension).

Eph activation does not only counteract neurotrophin signaling as shown in this study and by others [46]. In addition, EphA forward signaling was reported to suppress FGF signaling [46]. Thus, such a repressive function on ERK signaling and gene activity which in principle could be exerted by multiple Eph family members (see introduction) might modulate receptor tyrosine kinase signaling in many biological functions including organogenesis and tumorigenesis [11].

## Supporting Information

Fig. S1 **BDNF stimulated neurite branching is antagonized by Eph forward signaling.** Cultures were incubated with ephrin-A5 and BDNF at the indicated combinations for three days, followed by staining for F-actin (red) and microtubules (green). (A, B) Untreated wild-type neurons (A) protruded multiple neurites and neurite length was increased compared to SRF-deficient neurons (B). (C, D) BDNF alone enhanced neurite numbers in wild-type (arrows in C), but not *Srf* mutant neurons (D). (E, F) Ephrin-A5 did not alter neurite numbers in wild-type (E) or SRF-deficient (F) neurons. (G, H) Ephrin-A5 antagonized the BDNF-stimulated increase in neurite branching in wild-type neurons (G). In *Srf* mutant neurons, no modulation of neurite numbers by ephrin-A5 and BDNF co-application was observable (H). (I, J) Quantification of average neurite (I) and branch (J) number. Statistical significance was calculated in relation to wild-type/control treatment. (K) BDNF-mediated increase in neurite number requires MEK as revealed by U-0126 application, a MEK inhibitor. *, P<0.05; **, P<0.01; ***, P<0.001. Error bars represent s.d. Scale-bar (A-H)  =  50 µm.
(DOC)

Fig. S2 **Unlike ephrin-As, the repulsive guidance cue semaphorin-3A does not antagonize BDNF-meditated IEG induction.** Cortical neurons were stimulated for 20 mins with 2.5 ng/ml BDNF, supernatant of HEK293 cells transfected with either GFP (control; ctr.) or semaphorin 3A (sema3A) or BDNF together with sema3A. Subsequently, RNA was isolated and cDNA was subjected to qPCR using primers to IEGs indicated. Growth cone collapse experiments were performed in parallel to confirm repulsive activity of sema3A supernatants used in qPCR experiments. Numbers in bars indicate numbers of independent cultures used. The IEGs *Arc* (A), *c-fos* (B), *Egr1* (C) and *Egr2* (D) were induced by BDNF. Interestingly, *Arc* (A) and *Egr2* (D) were slightly induced by semaphorin 3A alone. Co-application of BDNF and sema3a resulted in robust induction of all IEGs tested to an extent comparable to BDNF alone. Thus, the repulsive guidance cue semaphorin 3A does not, unlike ephrin-A5, suppress BDNF-induced IEG mRNA levels.
(DOC)

Fig. S3 **Constitutively-active MEK1 (caMEK1) elevates P-ERK levels.** Neurons were electroporated with indicated amounts of a plasmid expressing caMEK-FLAG. After two days in culture, cell lysates were prepared and subjected to Western Blotting with antibodies indicated. P-Erk levels were increased by overexpression of caMEK compared to mock-electroporated neurons.
(DOC)

Fig. S4 **Alterations in cytoskeletal gene expression upon exposure to ephrins and BDNF.** Wild-type cortical cultures were treated for 20 minutes (A, C, E, G) or 16h (B, D, F, H) with guidance cues as depicted, followed by quantification of mRNA levels of indicated genes by qRT-PCR. (A, B) Tropomyosin1 (*Tpm1*) was up-regulated within 20 minutes by ephrin-A5 alone (A). 16h exposure to BDNF, ephrin-A5 alone and both together resulted in elevated *Tpm1* mRNA levels (B). (C, D) Tropomyosin2 (*Tpm2*) was, within this 20 minute stimulation, not obviously altered by any treatment (C). Contrastingly, after 16h of incubation, BDNF slightly and ephrin-A5 alone or both together increased more robustly *Tpm2* mRNA amounts (D). (E, F) Actinin1 (*Actn1*) was slightly, yet significantly, induced in wild-type neurons by ephrin-A5 and BDNF co-application, but none of the other treatments. With longer exposure, all three combinations of guidance cues elevated *Actn1* mRNA levels (F). (G, H) Within 20 minutes of incubation, dynein light chain (*Dnal1*) was not changed by any guidance cue (G). After 16 h of application, BDNF slightly and ephrin-A5 and both together more pronounced, elevated *Dnal1* mRNA abundance. *, P<0.05; **, P<0.01; ***, P<0.001. Error bars represent s.d.
(DOC)
