# Supplementary material for: Ephrin-A5 Suppresses Neurotrophin Evoked Neuronal Motility, ERK Activation and Gene Expression
Source: PLoS One. 2011 Oct 11;6(10):e26089. doi: 10.1371/journal.pone.0026089 (PMC3191169; doi:10.1371/journal.pone.0026089)
Supplement: Fig. S1 — BDNF stimulated neurite branching is antagonized by Eph forward signaling. Cultures were incubated with ephrin-A5 and BDNF at the indicated combinations for three days, followed by staining for F-actin (red) and microtubules (green). (A, B) Untreated wild-type neurons (A) protruded multiple neurites and neurite length was increased compared to SRF-deficient neurons (B). (C, D) BDNF alone enhanced neurite numbers in wild-type (arrows in C), but not Srf mutant neurons (D). (E, F) Ephrin-A5 did not alter neurite numbers in wild-type (E) or SRF-deficient (F) neurons. (G, H) Ephrin-A5 antagonized the BDNF-stimulated increase in neurite branching in wild-type neurons (G). In Srf mutant neurons, no modulation of neurite numbers by ephrin-A5 and BDNF co-application was observable (H). (I, J) Quantification of average neurite (I) and branch (J) number. Statistical significance was calculated in relation to wild-type/control treatment. (K) BDNF-mediated increase in neurite number requires MEK as revealed by U-0126 application, a MEK inhibitor. *, P<0.05; **, P<0.01; ***, P<0.001. Error bars represent s.d. Scale-bar (A-H) = 50 µm. (DOC) [file pone.0026089.s001.doc]

**Supplemental Figure 1**

**
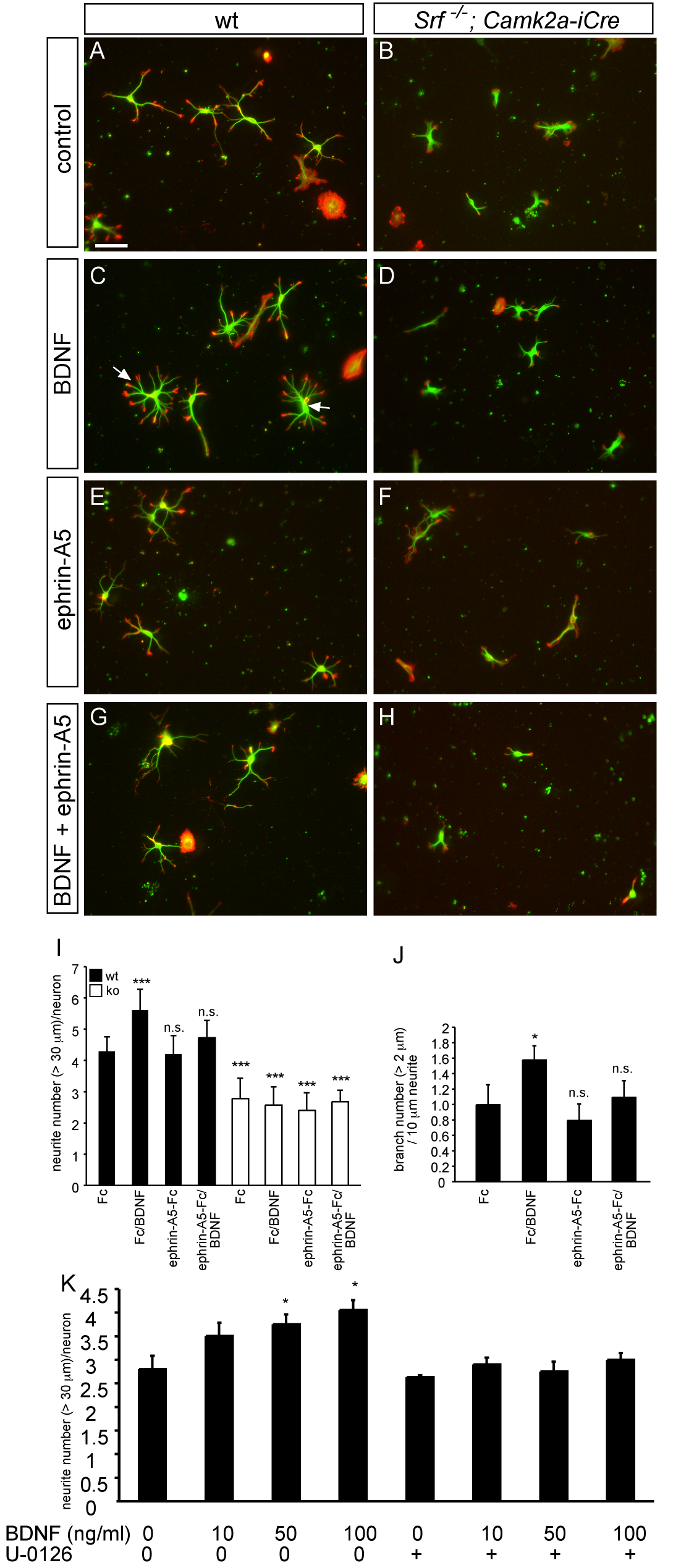
**

**BDNF stimulated neurite branching is antagonized by Eph forward signaling**

Cultures were incubated with ephrin-A5 and BDNF at the indicated combinations for three days, followed by staining for F-actin (red) and microtubules (green).

(A, B) Untreated wild-type neurons (A) protruded multiple neurites and neurite length was increased compared to SRF-deficient neurons (B).

(C, D) BDNF alone enhanced neurite numbers in wild-type (arrows in C), but not *Srf* mutant neurons (D).

(E, F) Ephrin-A5 did not alter neurite numbers in wild-type (E) or SRF-deficient (F) neurons.

(G, H) Ephrin-A5 antagonized the BDNF-stimulated increase in neurite branching in wild-type neurons (G). In *Srf* mutant neurons, no modulation of neurite numbers by ephrin-A5 and BDNF co-application was observable (H).

(I, J) Quantification of average neurite (I) and branch (J) number. Statistical significance was calculated in relation to wild-type/control treatment.

(K) BDNF-mediated increase in neurite number requires MEK as revealed by U-0126 application, a MEK inhibitor.

*, P < 0.05; **, P < 0.01; ***, P < 0.001. Error bars represent s.d..

Scale-bar (A-H) = 50 μm
