# Supplementary material for: Ephrin-A5 Suppresses Neurotrophin Evoked Neuronal Motility, ERK Activation and Gene Expression
Source: PLoS One. 2011 Oct 11;6(10):e26089. doi: 10.1371/journal.pone.0026089 (PMC3191169; doi:10.1371/journal.pone.0026089)
Supplement: Fig. S2 — Unlike ephrin-As, the repulsive guidance cue semaphorin-3A does not antagonize BDNF-meditated IEG induction. Cortical neurons were stimulated for 20 mins with 2.5 ng/ml BDNF, supernatant of HEK293 cells transfected with either GFP (control; ctr.) or semaphorin 3A (sema3A) or BDNF together with sema3A. Subsequently, RNA was isolated and cDNA was subjected to qPCR using primers to IEGs indicated. Growth cone collapse experiments were performed in parallel to confirm repulsive activity of sema3A supernatants used in qPCR experiments. Numbers in bars indicate numbers of independent cultures used. The IEGs Arc (A), c-fos (B), Egr1 (C) and Egr2 (D) were induced by BDNF. Interestingly, Arc (A) and Egr2 (D) were slightly induced by semaphorin 3A alone. Co-application of BDNF and sema3a resulted in robust induction of all IEGs tested to an extent comparable to BDNF alone. Thus, the repulsive guidance cue semaphorin 3A does not, unlike ephrin-A5, suppress BDNF-induced IEG mRNA levels. (DOC) [file pone.0026089.s002.doc]

# Supplemental Figure 2


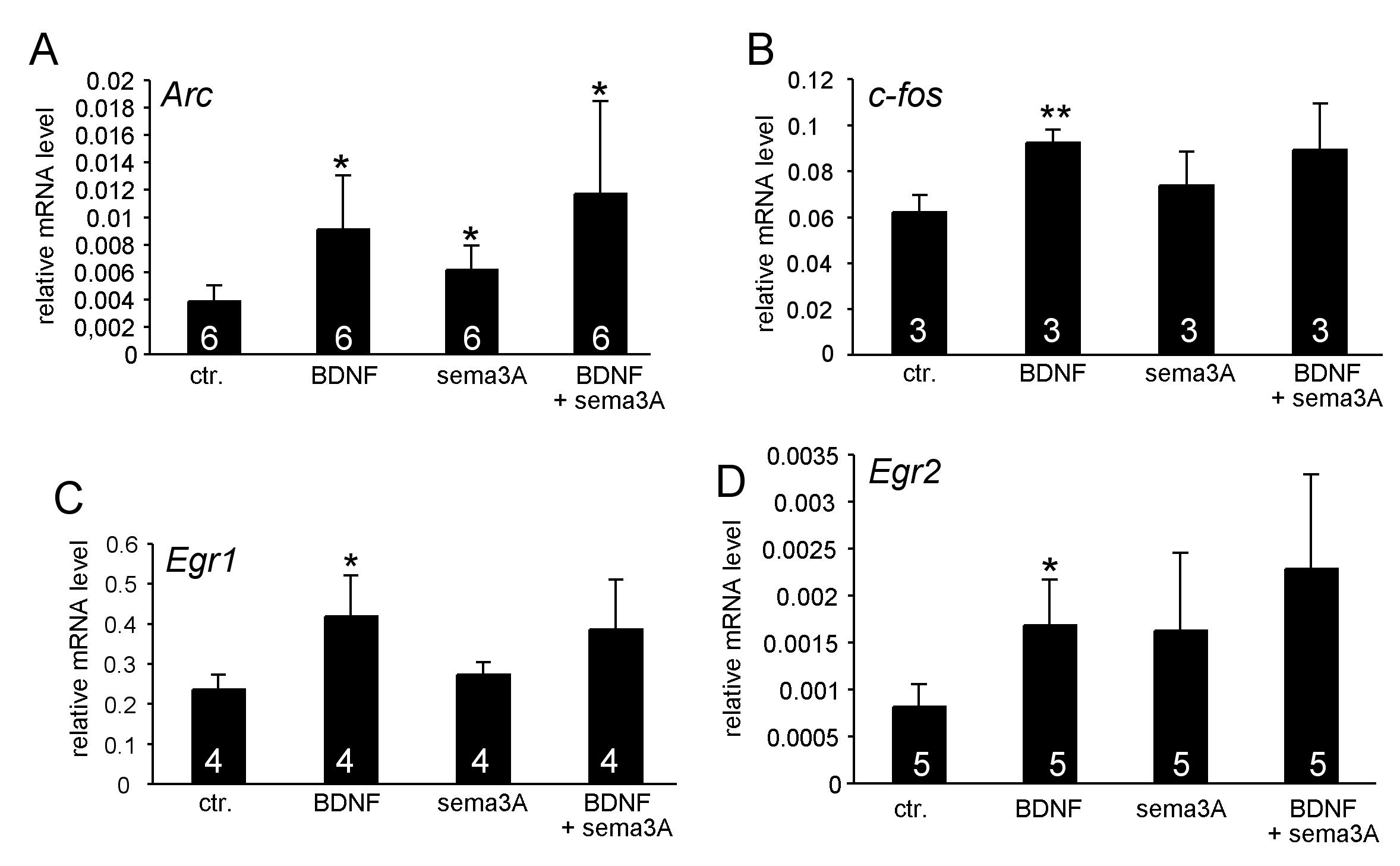


**Unlike ephrin-As, the repulsive guidance cue semaphorin-3A does not antagonize BDNF-meditated IEG induction**

Cortical neurons were stimulated for 20 mins with 2.5 ng/ ml BDNF, supernatant of HEK293 cells transfected with either GFP (control; ctr.) or semaphorin 3A (sema3A) or BDNF together with sema3A. Subsequently, RNA was isolated and cDNA was subjected to qPCR using primers to IEGs indicated. Growth cone collapse experiments were performed in parallel to confirm repulsive activity of sema3A supernatants used in qPCR experiments. Numbers in bars indicate numbers of independent cultures used.

The IEGs *Arc* (A), *c-fos* (B), *Egr1* (C) and *Egr2* (D) were induced by BDNF. Interestingly, *Arc* (A) and *Egr2* (D) were slightly induced by semaphorin 3A alone. Co-application of BDNF and sema3a resulted in robust induction of all IEGs tested to an extent comparable to BDNF alone. Thus, the repulsive guidance cue semaphorin 3A does not, unlike ephrin-A5, suppress BDNF-induced IEG mRNA levels.
