# Supplementary material for: Ephrin-A5 Suppresses Neurotrophin Evoked Neuronal Motility, ERK Activation and Gene Expression
Source: PLoS One. 2011 Oct 11;6(10):e26089. doi: 10.1371/journal.pone.0026089 (PMC3191169; doi:10.1371/journal.pone.0026089)
Supplement: Fig. S3 — Constitutively-active MEK1 (caMEK1) elevates P-ERK levels. Neurons were electroporated with indicated amounts of a plasmid expressing caMEK-FLAG. After two days in culture, cell lysates were prepared and subjected to Western Blotting with antibodies indicated. P-Erk levels were increased by overexpression of caMEK compared to mock-electroporated neurons. (DOC) [file pone.0026089.s003.doc]

# Supplemental Figure 3


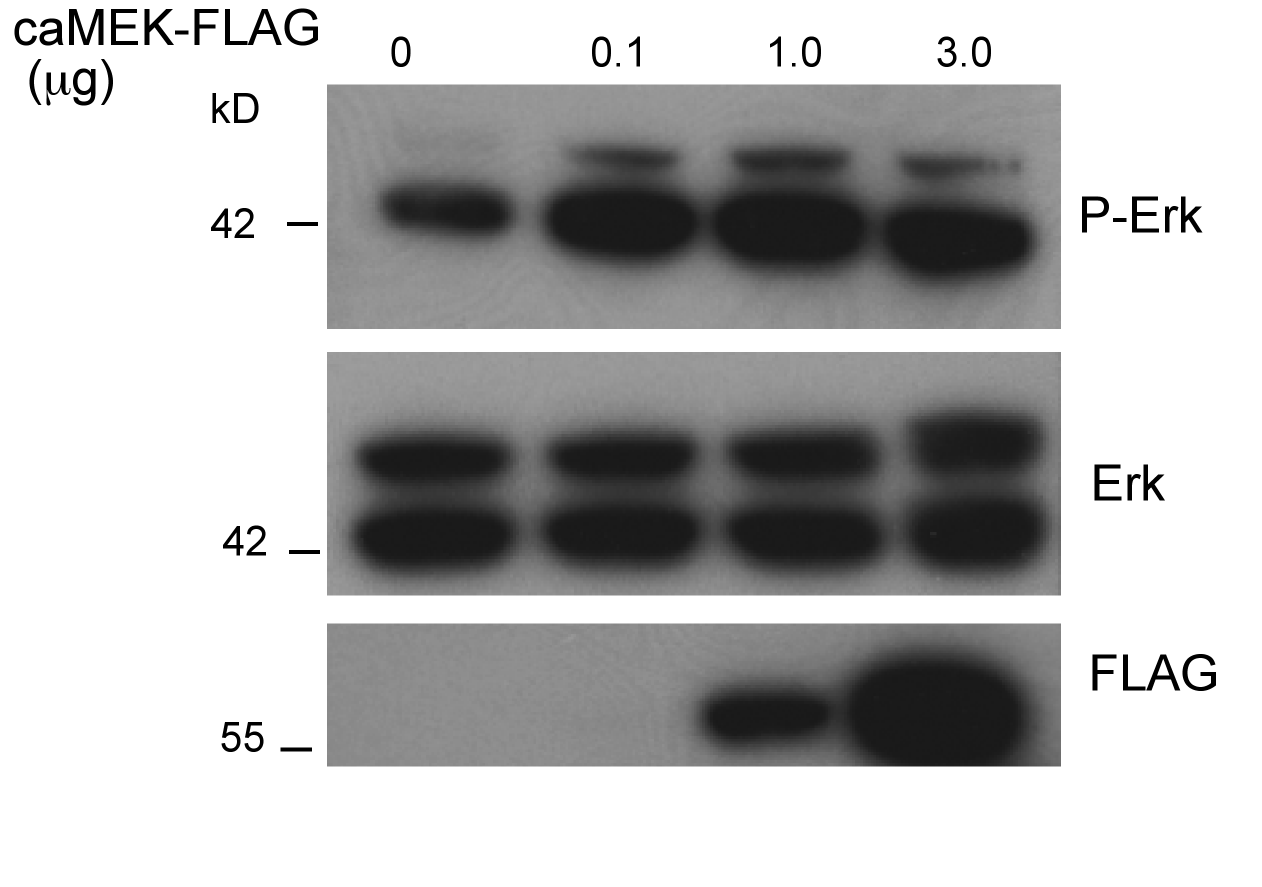


**Constitutively-active MEK1 (caMEK1) elevates P-ERK Levels**

Neurons were electroporated with indicated amounts of a plasmid expressing caMEK-FLAG. After two days in culture, cell lysates were prepared and subjected to Western Blotting with antibodies indicated. P-Erk levels were increased by overexpression of caMEK compared to mock-electroporated neurons.
