# Supplementary material for: Ephrin-A5 Suppresses Neurotrophin Evoked Neuronal Motility, ERK Activation and Gene Expression
Source: PLoS One. 2011 Oct 11;6(10):e26089. doi: 10.1371/journal.pone.0026089 (PMC3191169; doi:10.1371/journal.pone.0026089)
Supplement: Fig. S4 — Alterations in cytoskeletal gene expression upon exposure to ephrins and BDNF. Wild-type cortical cultures were treated for 20 minutes (A, C, E, G) or 16h (B, D, F, H) with guidance cues as depicted, followed by quantification of mRNA levels of indicated genes by qRT-PCR. (A, B) Tropomyosin1 (Tpm1) was up-regulated within 20 minutes by ephrin-A5 alone (A). 16h exposure to BDNF, ephrin-A5 alone and both together resulted in elevated Tpm1 mRNA levels (B). (C, D) Tropomyosin2 (Tpm2) was, within this 20 minute stimulation, not obviously altered by any treatment (C). Contrastingly, after 16h of incubation, BDNF slightly and ephrin-A5 alone or both together increased more robustly Tpm2 mRNA amounts (D). (E, F) Actinin1 (Actn1) was slightly, yet significantly, induced in wild-type neurons by ephrin-A5 and BDNF co-application, but none of the other treatments. With longer exposure, all three combinations of guidance cues elevated Actn1 mRNA levels (F). (G, H) Within 20 minutes of incubation, dynein light chain (Dnal1) was not changed by any guidance cue (G). After 16 h of application, BDNF slightly and ephrin-A5 and both together more pronounced, elevated Dnal1 mRNA abundance. *, P<0.05; **, P<0.01; ***, P<0.001. Error bars represent s.d. (DOC) [file pone.0026089.s004.doc]

# Supplemental Figure 4


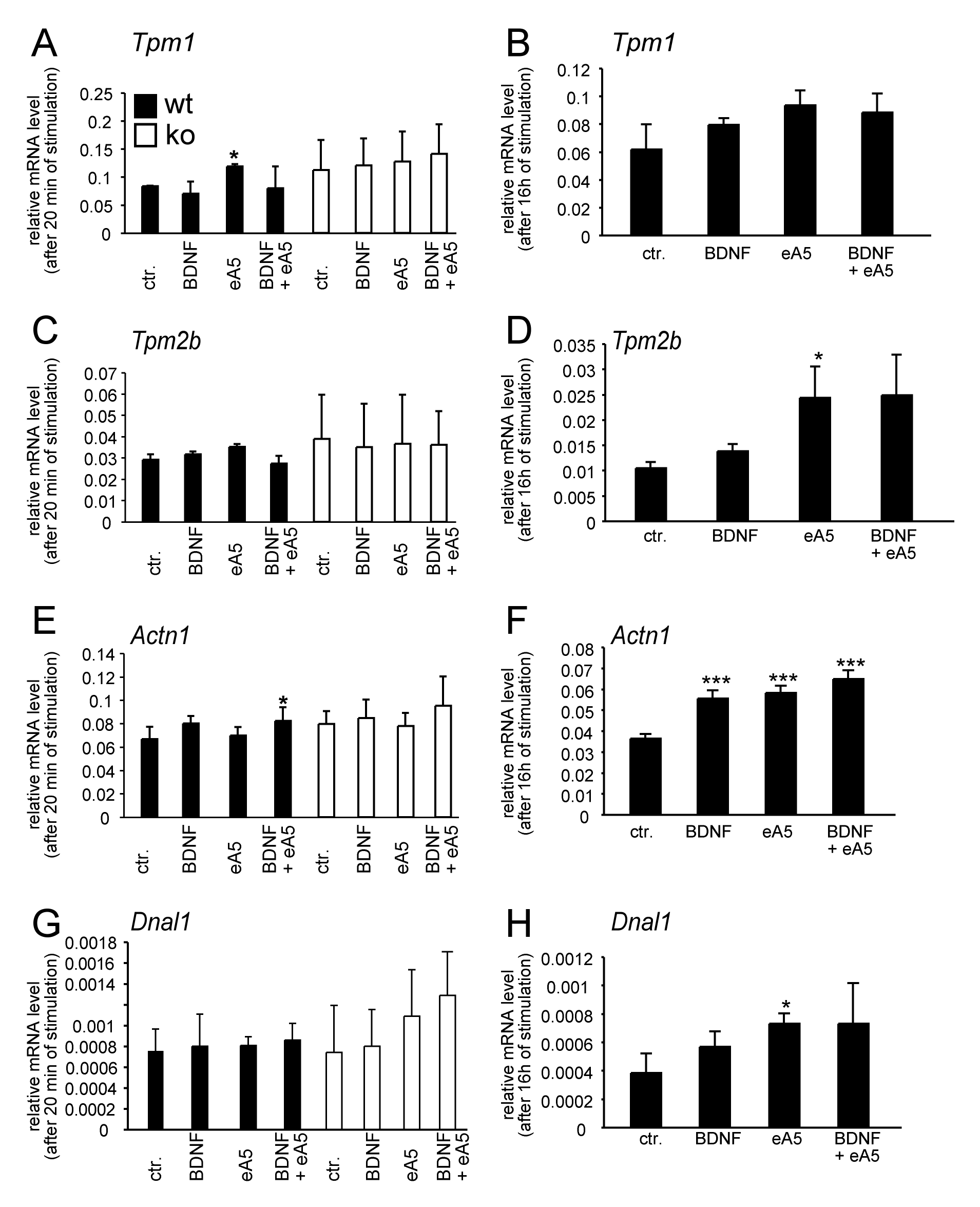


**Alterations in cytoskeletal gene expression upon exposure to ephrins and BDNF**

Wild-type cortical cultures were treated for 20 minutes (A, C, E, G) or 16h (B, D, F, H) with guidance cues as depicted, followed by quantification of mRNA levels of indicated genes by qRT-PCR.

(A, B) Tropomyosin1 (*Tpm1*) was up-regulated within 20 minutes by ephrin-A5 alone (A). 16h exposure to BDNF, ephrin-A5 alone and both together resulted in elevated *Tpm1* mRNA levels (B)

(C, D) Tropomyosin2 (*Tpm2*) was, within this 20 minute stimulation, not obviously altered by any treatment (C). Contrastingly, after 16h of incubation, BDNF slightly and ephrin-A5 alone or both together increased more robustly *Tpm2* mRNA amounts (D).

(E, F) Actinin1 (*Actn1*) was slightly, yet significantly, induced in wild-type neurons by ephrin-A5 and BDNF co-application, but none of the other treatments. With longer exposure, all three combinations of guidance cues elevated *Actn1* mRNA levels (F).

(G, H) Within 20 minutes of incubation, dynein light chain (*Dnal1*) was not changed by any guidance cue (G). After 16 h of application, BDNF slightly and ephrin-A5 and both together more pronounced, elevated *Dnal1* mRNA abundance.

*, P < 0.05; **, P < 0.01; ***, P < 0.001. Error bars represent s.d..
